# JinqiJiangtang tablets for pre-diabetes: A randomized, double-blind and placebo-controlled clinical trial

**DOI:** 10.1038/s41598-017-11583-5

**Published:** 2017-09-11

**Authors:** Hui Wang, Liping Guo, Hongcai Shang, Ming Ren, Xuemei Wang, Dehui Wang, Jianzong Chen, Shuanglei Li, Liming Chen, Yue Wang, Zhi Liu, Jingbo Zhai, Yuzhen Song, Hongbo Cao, Junhua Zhang, Chunxiang Liu, Xiao Sun, Da Huo, Wei Mu, Li Zhang, Wenke Zheng, Xiaoyan Yan, Chen Yao

**Affiliations:** 1 Evidence-based Medicine Center, Institute of Traditional Chinese Medicine,Tianjin University of Traditional Chinese Medicine, 312 Anshan Western Road, Nankai District, Tianjin, 300193 China; 2 Key Laboratory of Chinese Internal Medicine of Ministry of Education and Beijing, Dongzhimen Hospital, Beijing University of Chinese Medicine, Beijing, 100700 China; 3 Department of Integrated Chinese and Western Medicine, The First Hospital of Peking University, Xishiku Street, Xichen district, Beijing, 100034 China; 4 Department of Endocrinology, The Second Affiliated Hospital of Tianjin University of TCM,Zhenli Street, Hebei District, Tianjin, 300150 China; 5 Department of TCM, Xijing hospital, Changlexilu Street, Xi’an, 710032 China; 6 Department of Endocrinology, The First Affiliated Hospital of Guangxi College of TCM, Yuanhu Street, Nanning, 530023 China; 7 Department of Endocrinology, Metabolic Disease Hospital of Tianjin Medical University, Pingjiang Street, Hexi District, Tianjin, 300211 China; 8 Inner Mongolia people’s hospital, zhaowudastreet, Hohhot, 010017 China; 9 Tangshan Chinese Medicine Hospital, Kang Zhuang Street, Tangshan, 063000 China; 10 Clinical institute of Peking University, Xishiku Street, Xicheng District, Beijing, 100034 China

## Abstract

This study observed the efficacy and safety of JinqiJiangtang tablets (JQJT tablets, a traditional Chinese patent medicine) for pre-diabetes. Four hundred patients with pre-diabetes at five centres were treated for 12months and followed for an additional 12months to investigate the preventative effects of JQJT tablets (Registration ID: ChiCTR-PRC-09000401). The incidence rate of diabetes mellitus was the primary endpoint. The risk of converting from pre-diabetes to diabetes was 0.58-fold less in the JQJT tablets group than in the placebo group [HR (95% CI): 0.58 (0.384, 0.876), P = 0.010]. Furthermore, the probability of achieving normalized blood glucose was 1.41-fold greater in the JQJT tablets group than in the placebo group [HR (95% CI): 1.41 (1.002, 1.996), P = 0.0049]. ITT analysis revealed that the incidence of diabetes upon treatment completion was 16.5% in the JQJT tablets group compared with 28.9% in the control group. The percentage of patients with normalized blood glucose upon 12-month intervention was 41.8% in the JQJT tablets group compared with 27.8% in the control group. JQJT tablets could be an effective intervention for preventative treatment of Type 2 diabetes mellitus.

## Introduction

Diabetes is a major threat to global health. The incidence of diabetes is rapidly increasing worldwide, and many pre-diabetic patients are converting to diabetes. The most recent estimate by the International Diabetes Federation (IDF) is that 8.3% of adults, representing 382 million people globally, have diabetes, and the number is predicted to rise beyond 592 million within 25 years. Annually, 510 million of the world’s population dies from diabetes-related diseases, accounting for 8.39% of all deaths^1^. Furthermore, diabetes cost at least 548 billion US health expenditure dollars in 2013—11% of total health spending on adults. Despite the heavy social and financial burdens that diabetes incurs^1^, public awareness of the disease and its potential harms and treatment options is low^2^. Approximately half of patients suffering from diabetes are not aware of their illness^3^.

Pre-diabetes is a health condition in which a person’s blood sugar is higher than normal but not yet not high enough for a medical diagnosis of diabetes. The U.S. Centers for Disease Control (CDC) estimated that 79 million Americans (approximately 35% of the population over 20 years old) have pre-diabetes^4^, and up to 70% of these patients eventually progress to diabetes^5^.The average risk of developing diabetes is approximately 5–10% per year in individuals with impaired fasting glucose (IFG) or impaired glucose tolerance (IGT) compared with approximately 0.7% per year in normoglycemic individuals^6^. In 2013, the global prevalence of IGT was estimated at 316 million—a number expected to rise to 471 million by 2035^7^.Facing this reality, the WHO believes that prevention is the best cure, and early prevention is required for the pre-diabetes population. Pre-diabetes is a critical state in which abnormal blood sugar could be restored to normal levels if adequate intervention is initiated. Traditional Chinese medical practice has a long-standing tradition of prioritizing disease prevention over treatment. *The Yellow Emperor’s Classic of Internal Medicine* (HuangdiNeijing,in Chinese pinyin, compiled approximately 770–221 B.C.) recorded therapeutic concepts such as“medical practitioners of high proficiency intervene before one fallsill” and “prevention is the best treatment”. Currently, treatment options for pre-diabetes are scarce. We believe that traditional Chinese therapy may provide a promising means of diabetes prevention.

Developed by the Institute of Materia Medica of the Chinese Academy of Medical Sciences, JQJT tablets are the first new Chinese patent drug to be approved by the Chinese Food and Drug Administration(CFDA) for diabetes treatment^8^.JQJT tablets are composed of berberine, astragalus and honeysuckle, and the main functions include clearing heat, replenishing qi and engendering body fluids to check thirst. The multiple components of JQJT tablets have been shown in laboratory settings to improve insulin resistance by regulating glucose and lipid metabolism, inhibiting oxidation and boosting immune function^9^.

**Figure 1:**
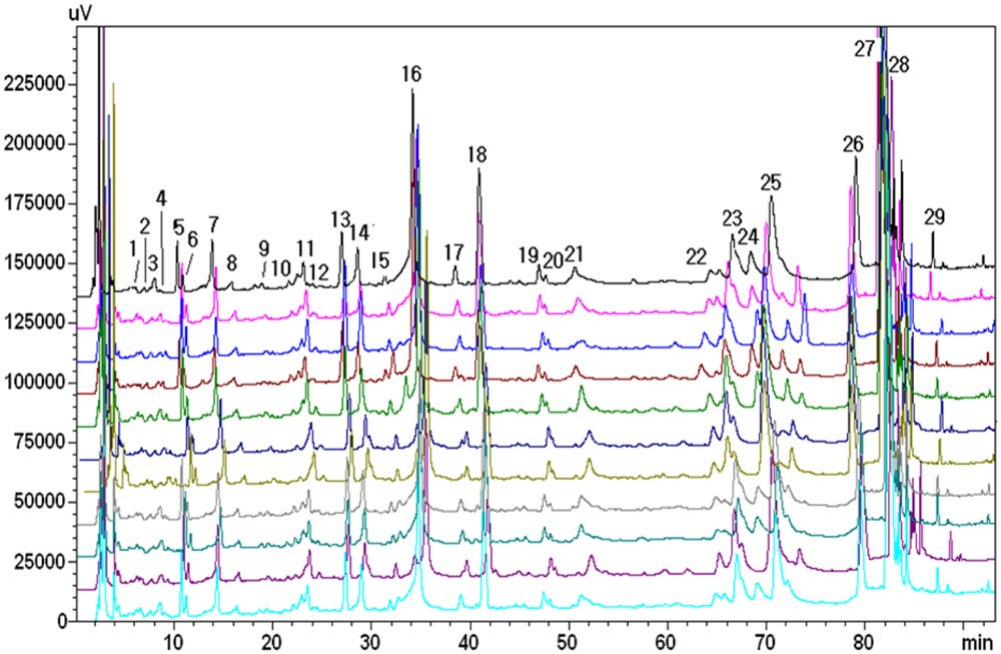
Fingerprints of 11 batches of JQJT tablets^10^.

The cultivation and manufacture of raw materials for JQJT tablets is in accordance with the Good Agriculture Practice and Good Manufacturing Practice Standards. Our group found that the HPLC determination of 11 different batches of JQJT tablets samples (see Fig. 1) exhibited characteristic co-possessing peaks^10^, a proof of the consistency of drug quality. Of all the co-possessing peaks, peak No. 2 was identified as chlorogenic acid, No. 5 as rutin, No. 15 as 4,5 -dicaffeoylquinic acid, No. 17 as luteoloside, No. 23 as Jatrorrhizine Hydrochloride, No. 27 as palmatine and No. 28 as berberine hydrochloride. No. 28 represents the internal reference peak.

In this study, we conducted a rigorously designed controlled clinical trial to investigate the efficacy and safety of JQJT tablets for pre-diabetes during a 12-month treatment period and an additional 12-month follow-up period.

## Materials and Methods

Detailed descriptions of the design, intervention, randomization, allocation concealment, blinding, sample size calculation, subject recruitment, and outcome measures have been published in a study protocol in Trials^11^.

### Study Design

This was a randomized, double-blind and placebo-controlled clinical study. The entire study was conducted according to the Declaration of Helsinki and the International Conference on Harmonization Tripartite Guideline on Good Clinical Practice. The study was approved by the ethics committees of the Chinese clinical trial registry(No. ChiECRCT-20090009). Informed consent was obtained before the study. Trial registration no. was ChiCTR-PRC-09000401 (Date of Registration:2009–05–08).

Tianjin University of Traditional Chinese Medicine (TCM) was held responsible for the organization and coordination of the trial sites and staffs. The participating institutions included the First Hospital of Peking University, the Second Affiliated Hospital of Tianjin University of TCM, the Metabolic Diseases Hospital of Tianjin Medical University, the First Affiliated Hospital of Guangxi Medical University and XiJing Hospital.

### Study population

The inclusion criteria were formulated according to the 2008 Guidelines for Diabetes Treatment developed by the American Diabetes Association. Impaired fasting glucose (IFG) (FPG5.6–6.9 mmol/L) and/or impaired glucose tolerance (IGT) (FPG <5.6 mmol/L and OGTT 2-hPG 7.8–11.1 mmol/L).

### Inclusion criteria

- Fulfilment of pre-diabetes diagnosticcriteria;
- Age18–75 years old;
- Completed and submitted informed consent form;

### Exclusion criteria

- History of diabetes (except gestational diabetes);
- Cardiovascular event;
- Impaired hepatic and renal function: AST and/or ALT 2-fold the upper limit of normal or above; same for creatinine. Urine protein >  +  + and/or haematuria.
- Fasting triglycerides ≥10 mmol/L;
- Endocrine disease, such as hyperthyroidism, autoallergic disease, cancer or other serious fatal illness;
- Former use of glucocorticoids, β receptor blockers,thiazide diuretics and nicotinic acid;
- Pregnancy, planned pregnancy and lactating women;
- Suffering from mental diseases or non-cooperating patients;
- Participating in other clinical trial within the last two weeks;
- Refusal to provide consent for the study.

### Intervention

Participants were administered 7 JQJT tablets or placebo twice daily time before meals for 12 months and were followed for an additional 12 months. The experimental and control drugs were manufactured by Zhongxin Pharma·Tianjin Longshunrong Pharmaceutical Factory. Both groups received lifestyle intervention as a basic treatment(health education according to the missionary handbook). Participants were required to make monthly visit to the trial site and visits in the 2nd, 6th and 12th month of the follow-up period.

The use of anti-diabetic drugs and drugs that may affect blood sugar levels, such as enalapril maleate folate, prednisone and furosemide, were forbidden. Other medications or therapies required to treat concomitant diseases were allowed during the study for ethical considerations. Information such as the name of the drug or therapy, actual dosage, and dosing frequency were well-documented.

### Randomization

Participants were allocated randomly into one of the two groups with a 1:1 allocation. An independent data centre named Interact Voice Responding System (IVRS) was used to complete the randomization according to a random sequence table (generated bySAS8.2). The randomized treatment assignments were sealed in opaque envelopes and controlled by the appointed individual who was not involved in this trial.

### Blinding

Simulated agents of JQJT tablets were made to ensure double-blinding in this study. Simulated agents of JQJT tablets had the same appearance, shape, colour and packaging as did JQJT tablets; thus, the researchers and participants did not know the identity of the medication. Blinding was maintained amongst the investigators and patients to guarantee the authenticity of the statistical outcomes and final report.

### Outcome measures

Primary outcome was incidence rate of diabetes mellitus. Secondary outcomes included the percentage of participants with normalized blood glucose(FPG < 5.6 mmol/L and OGTT 2-hPG < 7.8 mmol/L),oral glucose tolerance test and haemoglobin A1c (HBA1c) levels.

Outcome measures for safety evaluation included the incidence of adverse events, vital signs and related biochemical outcomes. Details of any adverse reactions or events were recorded in the case report form, including the time of occurrence, the severity and duration of adverse reactions or events, any intervention prescribed and the patients’ responses.

### Statistical analysis

We used the SAS 9.3 software package for data analysis. Quantitative data such as age, height and weight were compared using the t-test or the Wilcoxon rank-sum test, and categorical variables such as sex, medical history and drug-taking history were compared using the chi-square test or exact test to check baseline comparability.

We built a Cox’s proportional hazards regression model to calculate and compare the hazard ratio of having blood glucose restored to normal and of developing diabetes after treatment between the two groups. The FPG (fasting plasma glucose) levels, 2-h postprandial blood glucose (PBG) levels, OGTT levels and HBA1c levels were compared between the groups using the t-test and Wilcoxon rank sum test. All statistical tests were two-sided, and statistical significance was defined as P values less than or equal to 0.05.The first patient was enrolled in March 2010, the last patient was enrolled in January 2012, and the data analysis was performed in September 2013.

## Results

**Figure 2:**
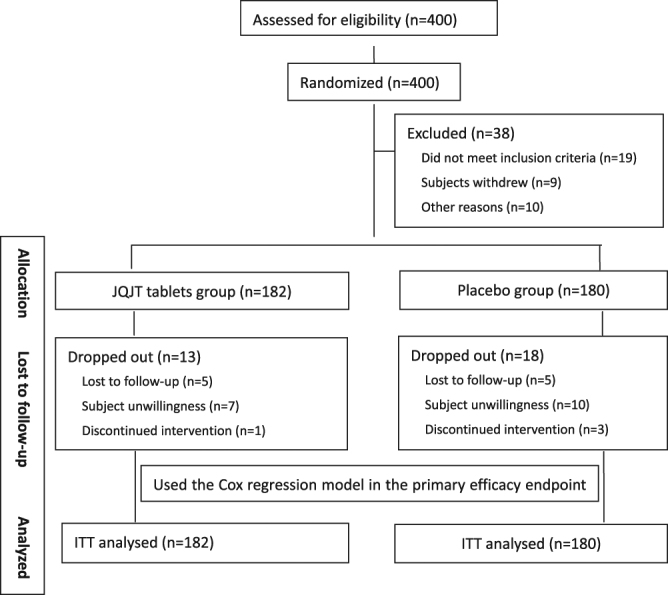
Study flowchart.

A total of 400 patients were screened for eligibility. Four hundred patients entered the randomization phase, and 38 were excluded, leading to a final total of 182 participants in the treatment group and 180 in the control group (See Fig. 2 for the flow of the study).

### Baseline characteristics

The baseline characteristics of the study groups are presented in Table 1. The distributions of the demographic and clinical characteristics between the two groups were reasonably well-balanced, except that the mean age in the placebo group was younger than that in the JQJT tablets group (53.49 ± 8.85 vs. 55.49 ± 8.61, P = 0.036).

**Table 1:** Baseline data for trial participants.

Characteristic	JQJT tablets group N = 182	Placebo group N = 180	P value
Age^*^, years	55.49 ± 8.61	53.49 ± 8.85	0.036
Male, n (%)	90(49.6)	82(45.6)	0.458
Ethnic group-Han, n (%)	179(98.4)	177(98.3)	1.000
Height, cm	165.35 ± 8.07	165.19 ± 7.65	0.837
Weight, kg	68.06 ± 12.08	67.49 ± 11.21	0.951
Concomitant disease - YES, n (%)	45(24.7)	48(26.7)	0.673
Medication history -YES, n (%)	38(21.0)	35(19.4)	0.714
Respiratory rate, bpm	17.63 ± 1.08	17.57 ± 1.11	0.426
Pulse, bpm	72.47 ± 6.63	72.28 ± 7.46	0.923
Heart rate, bpm	72.50 ± 6.63	72.29 ± 7.46	0.948
SBP, mmHg	125.10 ± 9.54	123.74 ± 9.91	0.357
DBP, mmHg	77.48 ± 6.54	76.47 ± 7.73	0.358
Waistline, cm	85.29 ± 7.92	85.08 ± 8.92	0.932
Hipline, cm	98.36 ± 9.54	98.61 ± 9.48	0.944
OGTT –0min, mmol/L	5.97 ± 0.64	5.96 ± 0.69	0.832
OGTT – 120 min, mmol/L	8.91 ± 1.29	8.98 ± 1.44	0.292
Ins –0 min, mU/L	17.22 ± 22.88	16.89 ± 20.69	0.851
Ins –120 min, mU/L	122.57 ± 228.51	112.70 ± 184.83	0.260
HbA1c, %	6.21 ± 0.59	6.23 ± 0.61	0.679
TC, mmol/L	4.99 ± 0.86	4.98 ± 0.91	0.912
TG, mmol/L	1.69 ± 0.89	1.84 ± 1.14	0.347
HDL, mmol/L	1.35 ± 0.34	1.34 ± 0.37	0.852
LDL, mmol/L	2.89 ± 0.76	2.85 ± 0.70	0.567

^a^Note: Plus–minus values are means ± SD unless otherwise noted.

The “concomitant diseases”primarily included hypertension,hyperlipidaemia or coronary heart disease.

“Medication history”primarily included calcium channel blockers, angiotensin-converting enzyme inhibitors, angiotensin receptor blocker, lipid lowering drugs and antiplatelet drugs.

### Primary outcome

#### The risk of converting from pre-diabetes to diabetes over 24 months

**Figure 3:**
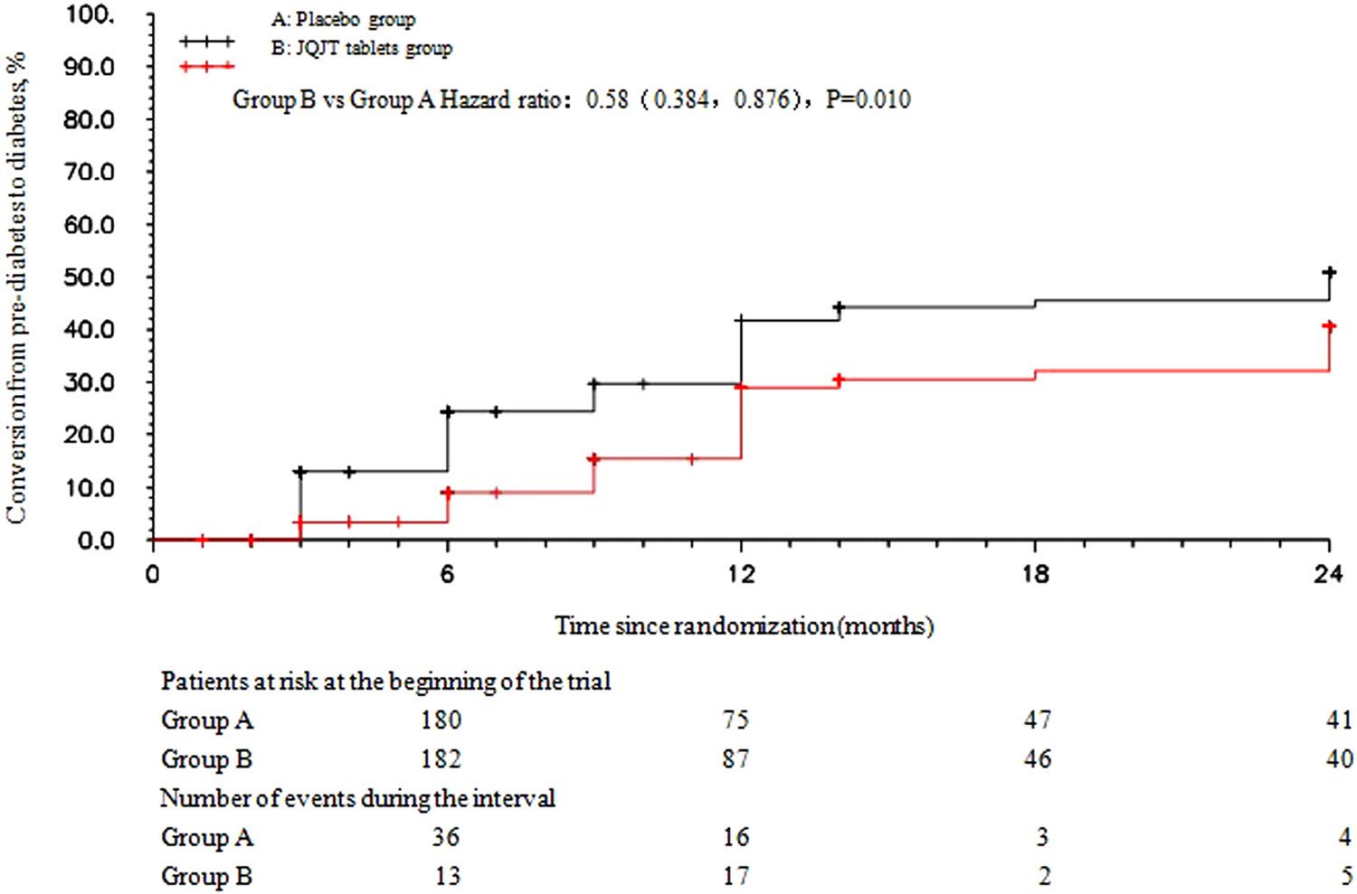
Risk curve depicting the risk of developing diabetes over 24 months

The risk of converting from pre-diabetes to diabetes was 0.58-fold less in the JQJT tablets group than in the placebo group [HR (95% CI): 0.58 (0.384, 0.876), P = 0.010] (Fig. 3).

### Secondary outcomes

**Figure 4:**
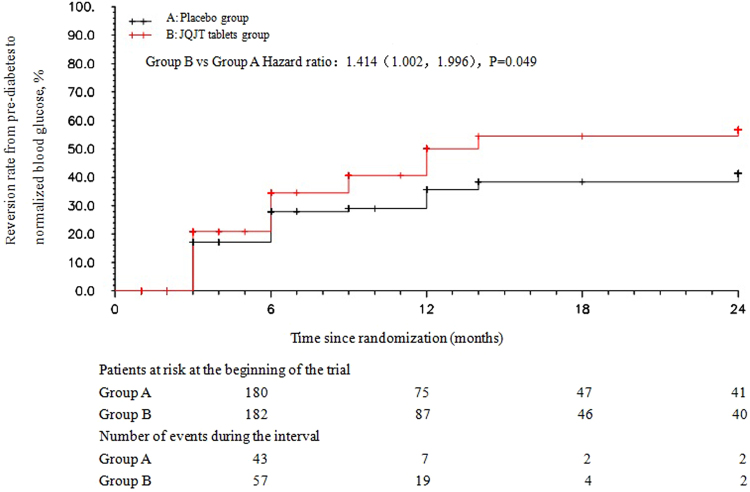
Risk curve of the probability of achieving normalized blood glucose levels over 24 months.

The probability of achieving normalized blood glucose was1.41-folds greater in the JQJT tablets group than in the placebo group [HR (95% CI): 1.41 (1.002, 1.996), P = 0.0049] (Fig. 4).

### Incidence rate of diabetes mellitus

The JQJT tablets group exhibited a significantly smaller increase in the proportion of patients developing diabetes compared with the placebo group (16.5%vs. 28.9% at month12, P = 0.005; 20. 3% vs. 32. 8% at month 24, P = 0.007).

At month 3, 6 patients developed new-onset diabetes in the JQJT tablets group, and 22 patients developed new-onset diabetes in the placebo group. The numbers of patients developing new-onset diabetes in JQJT and placebo groups in the following months were as follows: 7 and 14 at month 6, 6 and 5 at month 9, and 11 and 11 at month 12, respectively.

### The rate of normalization of blood glucose

JQJT tablets enhanced the rate of blood glucose normalization by 14% (41.8% for the JQJT tablets group vs. 27.8% for the placebo group, P = 0.005) upon completion of the 12-month treatment. The proportion increased to 15.1% at month 24, with 45.1% of pre-diabetic patients in the JQJT tablets group vs. 30.0% of patients in the placebo group (P = 0.003) achieving normalized blood glucose levels.

For the restoration of blood glucose, the number of pre-diabetic patients with normalized blood glucose was 36 in the JQJT tablets group vs. 29 in the placebo group at month 3; 21 vs. 14 at month 6;8 vs. 1 at month 9;and 11 vs. 6 at month 12, respectively.

### OGTT and HBA1c levels

**Figure 5:**
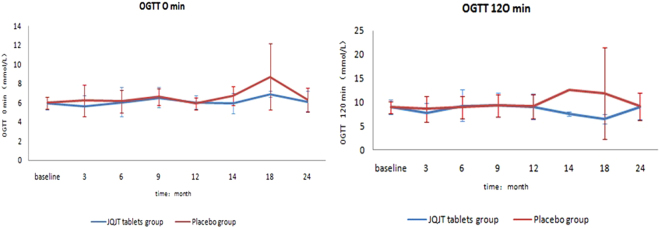
Changes in OGTT at 0 min and 120 min over 24months.

Blood was at various intervals to measure glucose levels, including before the OGTT began and two hours after the beverage was consumed. The FPG and the 120-min OGTT glucose levels were measured and compared between groups. As shown in Fig. 5, The JQJT tablets group exhibited lower mean FPG levels at all visit points except for month 12, when the placebo group exhibited lower FPG, although the difference was less than 0.13.These results suggest that JQJT tablets help decrease FPG levels in pre-diabetic patients. However, the mean 120-min OGTT glucose levels fluctuated between 6.43 and 9.41 in the JQJT tablets group, indicating poor or no effects on2-hPBG.

**Figure 6:**
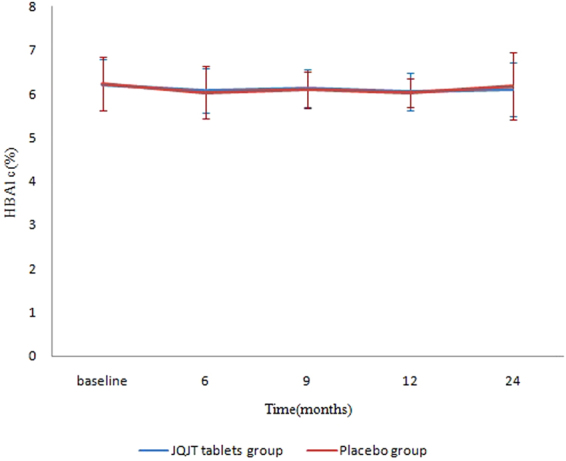
Change in HBA1c values over 24 months.

Moreover, we measured the HBA1c levels (Fig. 6), calculated changes from baseline, and performed within and between-group comparisons.Our research detected nostatistically meaningful difference in the HBA1c level between groups and for all visit points. Within-group analysis revealed that the HBA1c level dropped significantly from baseline. However, the between-group comparison suggested that changes from baseline were not statistically significant between groups.Thus, we concluded that JQJT tablets had no effects on HBA1c in the pre-diabetic population, likely because the HBA1c level is a stable indicator that is not prone to influence.The group-by-time interaction was not statistically significant for OGTT at 0 min (F = 0.54, P = 0.6663) or at 120 min (F = 1.56, P = 0.2654).

### Safety analysis

The safety of the investigational drugs was assessed based on the safety set. A total of 8 cases of adverse events were reported, including 5 cases in the placebo group and 3 in the JQJT tablets group.The adverse events included 5 cases of drug allergy(gastric upset), dizziness and chest pain,renal dysfunction, digestive upset,and headache in placebo group. The 3 cases in the JQJT tablets group included acute pneumonia, increased urine volume and bloating and qualitatively increased urine protein (++).The incidence of adverse events was 2.8% and 1.7% in the placebo and JQJT groups, respectively. The frequencies of these events did not differ significantly between the two groups (P = 0.501).There was no significant difference between the groups.

## Discussion

Research suggests that the pre-diabetic population is at high risk of progressing to diabetes mellitus and of experiencing adverse cardiovascular events such as myocardial infarction, stroke and cardiovascular death^12^. Furthermore, prediabetic patients are prone to nephropathy, neuropathy and retinopathy later in life^5^.Adequate preventative intervention could effectively retard disease progression and reduce the incidence of relevant comorbidities. Previous studies have demonstrated^13^ that 6-year lifestyle modification significantly decreases cardiovascular deaths(11.9% for the intervention vs. 19.6% for the control group) and all-cause mortality (28.1% for the intervention vs. 38.4% for the control group), after following patients with IGT for 23 years.Traditional Chinese medical practice with its advantages of mind-body balancing and lifestyle adjustment hold promise for the prevention of diabetes mellitus and its complications^14^.

JQJT tablets are composed of three Chinese medicinal herbs, i.e., rhizomecoptidis, astragalus and honeysuckle, and the primary bioactive constituents of the formula have been identified to include alkaloids, organic acids, flavonoids and saponins^10^.Research has attributed the hypoglycaemic effects of JQJT tablets primarily to berberine^15^ from rhizome coptidis^16, 17^ and has determined that astragalus and honeysuckle play a supplementary role^9^. The mechanism underlying the hypoglycaemic action of berberine involves multiple targets and pathways^18^. Berberine can improve insulin receptor expression, promote insulin secretion, enhance insulin sensitivity^19^, promote glucose utilization in liver cells^20^, and lower FBG and the incidence of hepatic steatosis through mitochondrial inhibition and AMPK(Adenosine 5′-monophosphate (AMP)-activated protein kinase) activation^21^. Moreover, berberine decreases blood lipids levels by stabilizing low density lipoprotein receptor mRNA,acting on the 3′UTR region at the post-transcriptional level, and improving glucose and lipid metabolism^22, 23^.Astragalus contains isoflavones, astragalussaponin compounds and many trace elements and can promote metabolism,scavenge oxygen free radicals, lower blood sugar levels^24, 25^, and improve insulin sensitivity^26^. The saponins of astragalus membranaceus have been shown to improve insulin resistance^6^ and help enhance the hypoglycaemic effects of berberine^27^. The pharmacological actions of honeysuckle include lowering blood pressure, protecting pancreatic β cells, and lowering blood sugar^28^. The herb also possesses antioxidant, anti-inflammatory and immune-boosting effects^29–32^, which could be a contributing factor in ameliorating insulin resistance. The sugar alcohol elements of lonicera japonica could inhibit blood glucose increases during OGTTs in a dose-dependent manner^6^.

Our research into the hypoglycaemic effects of JQJT tablets revealed that it significantly increased AMPK phosphorylation,promoted AMPK activation, decreased fat synthesis and increased fatty acid oxidation by inhibiting the activity of acetyl-CoA carboxylase (ACC), fatty acid synthase (FAS) and other fatty acid synthase, restrained hormone-sensitive triglyceride lipase(HSL)lipolysis,up-regulated glucose transporter type 4 (GLUT-4)and insulin receptor substrate1 and 2 (IRS1/2) expression, enhanced glucose uptake in the skeletal muscle, and improved insulin resistancein diabetic mice^33^. One study reported JQJT tablets reduced blood sugar levels, improved glucose tolerance, lowered blood lactic acid levels, reduced sugar content in the skin and promoted hepaticglycogen synthesis in normal mice, alloxan mice, and rats and obese mice with spontaneous diabetes^34^. JQJT tablets have also been shown to reduce blood triglyceride levels, alleviate fatty liver, enhance humoural and cellular immunity, improve insulin resistance^35^ and help restore the body’s sensitivity to insulin^36^. Moreover, researchers noted that JQJT tablets can increase insulin sensitivity through activating the AMPK signalling pathway, thus enhancing β cell function^6^.

With respect to the understanding and handling of pre-diabetes, Chinese medicine has abundant experience, as recorded in its profound historical texts. Inspired by the TCM theory that “the best treatment is always prevention”, we conducted a placebo-controlled study to evaluate the effectiveness and safety of JQJT tablets for pre-diabetes. Our study found that JQJT tablets effectively reduced the probability of developing diabetes and improved the likelihood of having normalized blood glucose after treatment in the pre-diabetic population. These findings added to the clinical body of evidence supporting the use of TCM for pre-diabetes.

Moreover, the action pattern of JQJT tablets for pre-diabetes had been identified. The onset of action took 3 months or less, and the clinical effects, with respect to decreasing the progression to diabetes and normalizing blood glucose, were most evident during the 12 months of medication. However, the hypoglycaemic effects became less prominent as the study proceeded and became undetectable during the 12-month follow-up period, indicating weak lasting effects.

Our research determined that JQJT tablets are effective in reducing FBG levels in pre-diabetes patients but exhibit poor or no effects on 2-h PBG. This finding is inconsistent with the results of our pre-clinical animal experiment, which demonstrated that JQJT tablets reduced FBG levels in both diabetic rats and insulin-resistant rats. In this clinical study, within-group analysis revealed that HBA1c levels decreased significantly from baseline for both the treatment and the control group. However, between-group comparisons suggested that the changes from baseline were not statistically significant, suggesting that JQJT tablets had no effect on HBA1c in the pre-diabetic population.

## Conclusions

In summary, JQJT tablets can effectively reduce the rate of conversion from pre-diabetes to T2DM and can improve blood sugar restoration in pre-diabetes patients.JQJT tablets could be an effective intervention for the preventative treatment of T2DM. Short-term use of JQJT tablets is recommended for the prevention of T2DM in pre-diabetes patients.
